# Taxonomic Distinctness of Demersal Fishes of the California Current: Moving Beyond Simple Measures of Diversity for Marine Ecosystem-Based Management

**DOI:** 10.1371/journal.pone.0010653

**Published:** 2010-05-17

**Authors:** Nick Tolimieri, Marti J. Anderson

**Affiliations:** 1 National Oceanic and Atmospheric Administration, Northwest Fisheries Science Center, Seattle, Washington, United States of America; 2 Institute of Information and Mathematical Sciences, Massey University, Auckland, New Zealand; NIWA, New Zealand

## Abstract

**Background:**

Large-scale patterns or trends in species diversity have long interested ecologists. The classic pattern is for diversity (e.g., species richness) to decrease with increasing latitude. Taxonomic distinctness is a diversity measure based on the relatedness of the species within a sample. Here we examined patterns of taxonomic distinctness in relation to latitude (ca. 32–48 °N) and depth (ca. 50–1220 m) for demersal fishes on the continental shelf and slope of the US Pacific coast.

**Methodology/Principal Findings:**

Both average taxonomic distinctness (AvTD) and variation in taxonomic distinctness (VarTD) changed with latitude and depth. AvTD was highest at approximately 500 m and lowest at around 200 m bottom depth. Latitudinal trends in AvTD were somewhat weaker and were depth-specific. AvTD increased with latitude on the shelf (50–150 m) but tended to decrease with latitude at deeper depths. Variation in taxonomic distinctness (VarTD) was highest around 300 m. As with AvTD, latitudinal trends in VarTD were depth-specific. On the shelf (50–150 m), VarTD increased with latitude, while in deeper areas the patterns were more complex. Closer inspection of the data showed that the number and distribution of species within the class Chondrichthyes were the primary drivers of the overall patterns seen in AvTD and VarTD, while the relatedness and distribution of species in the order Scorpaeniformes appeared to cause the relatively low observed values of AvTD at around 200 m.

**Conclusions/Significance:**

These trends contrast to some extent the patterns seen in earlier studies for species richness and evenness in demersal fishes along this coast and add to our understanding of diversity of the demersal fishes of the California Current.

## Introduction

Species are distributed neither uniformly nor randomly across the globe. Large-scale patterns in biodiversity have interested ecologists since at least the time of Wallace and Darwin [1], [2]. Understanding these large-scale patterns helps us to develop hypotheses regarding how communities and ecosystems are organized on both ecological and evolutionary time scales [2], [3]. For example, documenting patterns in biodiversity might also be important due to its potential relationship with ecosystem function [4]–[10]. Biodiversity is also a key concept for conservation and management, and is fundamental for ecosystem-based approaches [8], [11], [12]. Conserving biodiversity through the protection of species richness is often an implicit or explicit goal of many management and conservation strategies [8], [12]–[15].

One of the best known large-scale patterns of biodiversity is the decline in species richness from the equator towards the poles [2], [16]–[19]. This pattern has been observed in a range of taxa in many different environments—from terrestrial plants [20] to ants [21] to deep-water invertebrates [22] and marine fishes [23], [24], and numerous hypotheses have been proposed to explain the latitudinal gradient including: geographic area, evolutionary speed, geometric constraints and productivity [2]. In marine systems species richness is also related to depth, typically declining with depth, although it may be highest at intermediate depths [23]–[27]. Additionally, the relationship between biodiversity and large-scale geological features is important for the understanding of biogeography, which can help in designating management areas.

Biodiversity is, however, a complex concept and is much more intricate than just the total number of species in a given area [27], [28]. Workers have also examined patterns in species evenness [2], [22]–[24], but standard measures of either richness or evenness treat all species as equivalent in value in their contribution to diversity. That is, two species of fish are considered as diverse as one fish and one flatworm. Increasingly, the taxonomic relationships among species have been used to describe another dimension of biodiversity, allowing diversity to be considered within the context of deeper potential functional or evolutionary lineages [29]–[31].

Taxonomic distinctness quantifies diversity as the relatedness of the species within a sample, based on the distances between species in a classification tree [31]. Average taxonomic distinctness (Δ^+^ or AvTD) is the mean of all species-to-species distances through the tree for all pairs of species within a sample, and represents the taxonomic breadth of the sample. The variation in taxonomic distinctness (Λ^+^ or VarTD) is the variation in branch lengths among all pairs of species (it is not the variance of AvTD among samples), and is a measure of the irregularities and divergences in the distribution of branch lengths within a sample. Both indices are appealing because they are based on presence/absence data, and unlike many biodiversity measures, neither is affected by the number of species or the sampling effort [30], [32].

In the marine environment, taxonomic distinctness has been used as a tool to examine environmental degradation like the effects of trawling [33], fishing in general (through marine reserve status[34], pollution [35], and other anthropogenic impacts [36], [37]). In addition, general trends in taxonomic distinctness with latitude and depth have been examined for some marine invertebrate taxa [38]–[40] and demersal fishes [27], [41]. Previous studies of taxonomic distinctness in demersal fishes have been quite focused, limited to a depth range of <570 m and done within a single region spanning less than 1.5° of latitude [27], [41]). Even for studies of taxonomic distinctness for invertebrates spanning much larger latitudinal ranges, there have been limitations to inferences, due to incomplete data structures in the samples available for analysis (i.e., not all depth strata were sampled at all latitudes of interest, e.g., [39], [40]).

Here, we describe quantitatively how AvTD and VarTD varied with depth and latitude for demersal fishes on the continental shelf and slope of the western U.S.A. We also identified individual taxa that were primarily responsible for the observed overall patterns. This work is unprecedented for marine demersal fishes, covering a relatively broad range across temperate latitudes (ca. 32–48° N) and depth (ca. 50–1220 m), as well as having sufficient replication to allow investigation of potential interactions between these two gradients in their effects on taxonomic distinctness in demersal fishes.

## Materials and Methods

**Figure 1:**
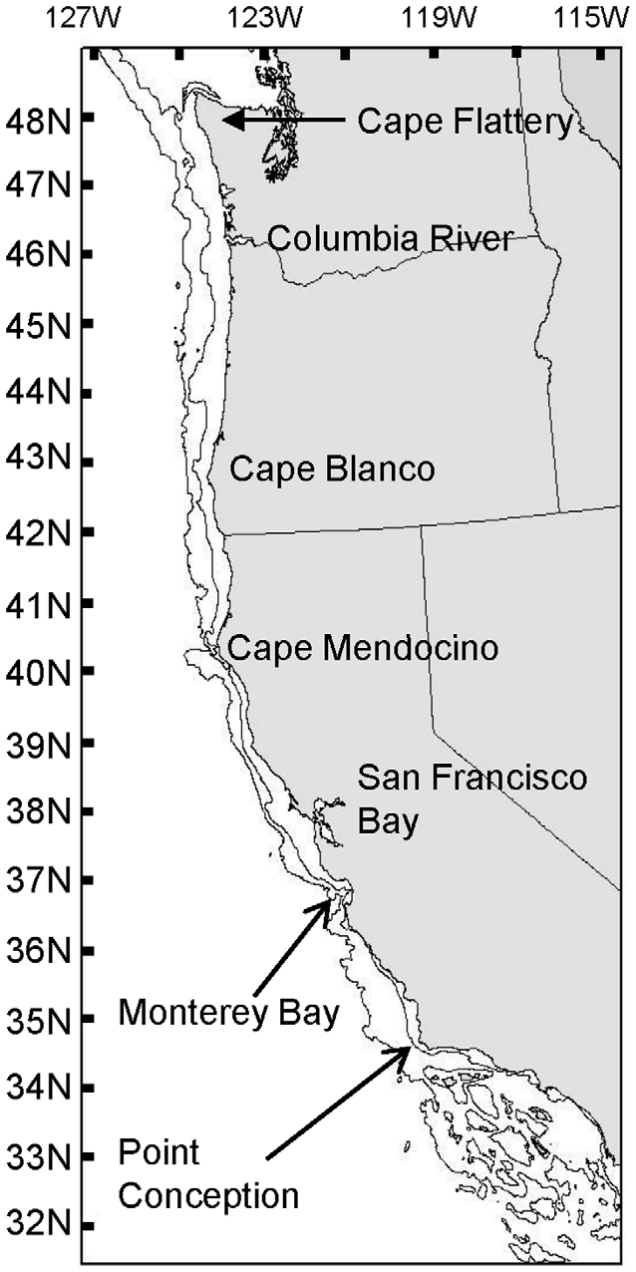
West coast of the USA. Bathymetry represents the 200 m and 1200 m depth contours.

We used data from the 1999–2002 Pacific West Coast Upper Continental Slope Trawl Survey [42] and the 2003 US West Coast Bottom Trawl Survey [43] to analyze trends in taxonomic distinctness with depth and latitude. The 1999–2002 survey was limited to the continental slope (184–1280 m) while the 2003 survey was expanded to included portions of the shelf (55–183 m). The trawl survey extends from 48°10′N to 32°30′N (Fig. 1). The trawls were carried out using Aberdeen style nets with a small mesh (5-cm stretched measure or less) liner in the cod-end. Trawl duration was approximately 15 minutes at 2.2 knots. Bottom contact and acoustic instruments were attached to the nets to record aspects of mechanical performance as well as gear depth. Catches were sorted to species level or closest taxonomic level. See Keller et al. [43] for details. We analyzed data consisting of only those taxa identified to species.

Although the trawl survey targets demersal species (fish typically associated with features of the ocean bottom), pelagic species are caught in the trawls as well. Preliminary analyses showed little difference between the results obtained with the full data set (all species) and those obtained with a reduced data set containing only demersal fishes. Therefore, we chose to analyze the full data set. However, the trawl survey does not sample complex, rocky habitat [44], [45], and the results of our analyses and our conclusions are limited to ‘trawlable habitat’.

### Diversity measures

We examined two measures of diversity: average taxonomic distinctness (AvTD or Δ^+^) and variation in taxonomic distinctness (VarTD or Λ^+^) [30]. AvTD is a measure of the taxonomic breadth of a sample. It utilizes presence/absence data and is calculated based on the taxonomic distance through a classification tree between every pair of species within a sample (here a trawl):
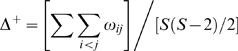
where 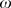 is the branch length between species pairs and *S* is the number of observed species in the sample. Here we used a standard Linnaean classification with 19 taxonomic levels [Table 1, 46]. We used the simple linear scaling [47] where the maximum distance through the tree is set at ω = 100. When branch lengths are un-weighted, the step between each taxonomic level in the tree is considered to be equal. However, all taxa were not defined to the same level of detail. For instance, some groups had defined tribes or subfamilies while others did not. Therefore, we weighted the branch lengths by the number of species having a definition at that level—essentially the amount of information in that taxonomic level. For example, 27% of species were defined at the level of tribe and 63% at the level of subfamily, but all species had definitions at the level of family (Table 1). AvTD is independent of sample size and the number of species in a sample [30], [31].

**Table 1:** Approximate weights and branch lengths used for all calculations of mean taxonomic distinctness and variation in taxonomic distinctness.

Taxon	Weight	Branch Length
Species	1.00	6
Genus	1.00	12
Tribe	0.27	14
Sub family	0.63	18
Family	1.00	24
Superfamily	0.33	26
Suborder	0.82	31
Order	1.00	37
Series	0.62	41
Superorder	0.93	46
Subdivision	0.99	52
Division	0.99	58
Subclass	0.99	64
Class	1.00	70
Subgrade	0.90	76
Grade	0.99	82
Superclass	1.00	88
Vertebrata	0.99	94
Subphylum	1.00	100

The weight is the proportion of species having a definition at that taxonomic level. Branch length is the resulting branch length within the taxonomic tree to that level after weighting.

VarTD is the variation in taxonomic distances between each pair of species about the AvTD value for that sample [32]:
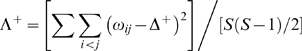
where ω is the branch length between pairs of species, *S* is the number of species observed in that sample and Δ^+^ is the average taxonomic distinctness for the sample as defined above. VarTD is also independent of sample size, number of species and the value of AvTD within a sample unit.

It is useful to examine both AvTD and VarTD because they capture independent aspects of the taxonomic diversity in a data set. Specifically, two taxonomic hierarchies can produce the same AvTD with different VarTD [31]. For example, a species list containing several different orders each with one genus and species, but also with some genera having many species, would give a high VarTD compared to a list (of equivalent AvTD) in which most species were from different families but within the same order [32]. Finally, the Chondrichthyes exhibited a strong influence on both AvTD and VarTD (see Results). Therefore, we also calculated the taxonomic distinctness measures for the Actinopterygii alone to examine taxonomic distinctness of this major taxon. Values of AvTD and VarTD were calculated for each trawl using PRIMER v6 [48].

### Data analysis

We used generalized additive models [GAMs], [ 49,50] to examine how AvTD and VarTD varied with latitude and depth because preliminary diagnostic plots suggested that relationships were not linear. For each measure we fit a two factor GAM (with identity link and Gaussian error distribution):
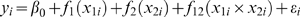
where *x*_1_ was depth, *x*_2_ was latitude, *β*_0_ was the intercept parameter, and *ε*_i_ were random normal errors with zero mean and a common variance The response variable *y* was either AvTD or VarTD. The smoothing functions *f*_1_ and *f*_2_ were thin plate regression splines [49]. Because depth and latitude were measured on different scales, we used a tensor product smooth (*f*_12_) of thin plate regression splines for the interaction term [51]. The optimal level of smoothing was chosen with general cross validation. Data were fit in R v2.10.0 using the package ‘mgcv’ [49], [52]. While the data set spans 55–1280 m, data were sparse in some of the deepest areas at some latitudes, so we limited the analyses to data collected from depths <1220 m to assure relatively even coverage across depths and latitudes. Diagnostic plots of residuals showed reasonable symmetry, but some deviation from normality in terms of kurtosis (Figure S1 & Figure S2). Although GAMs will be fairly robust to this kind of deviation, conclusions should nevertheless be taken with some caution.

To more specifically examine how AvTD and VarTD were related to latitude at particular depths (and vice versa), and to more fully investigate significant depth x latitude interactions (see Results), we conducted a second round of GAM (identity link, Gaussian distribution) analyses in which we binned data either by depth or by latitude zones. Here,
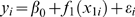
where *β*_0_ was the intercept parameter, *x* was depth within a specific latitude bin (or latitude within a specific depth bin), *f* was a thin plate regression spline and *ε_i_* were random normal errors with zero mean and a common variance.

We established the following five depth zones after examining the first analyses for AvTD (see Results): 50–150 m, 151–300 m, 301–600 m, 600–900 m, 900–1220 m. We binned data into four latitude zones based on approximate location of large-scale geographic features along the coastline: South of Point Conception, Point Conception to Cape Mendocino, Cape Mendocino to Cape Blanco, and North of Cape Blanco (Fig. 1).

When the degrees of freedom for the smoothed term were near their minimum value, we examined whether the trend was described sufficiently by a linear regression using Akaike's information criterion (AIC) and analysis of deviance between the two models.

### Importance of specific taxa

To identify which taxa influenced patterns of taxonomic distinctness the most, we used two complimentary steps: analysis of taxonomic trees and exclusion of specific taxa from the calculation of both AvTD and VarTD. First, we compared taxonomic trees across depth zones for the area between Point Conception and Cape Mendocino to examine how the branching patterns differed. We chose this region because here the r^2^ for depth was strongest for AvTD and high for VarTD (see results). We used the same depth strata as in previous analyses. We calculated the frequency of occurrence for each species in each depth x latitude bin and constructed trees for each bin that comprised those species which occurred in at least 50% of the hauls for that bin. These ‘50% trees’ do not completely describe the classification tree for the five depth zones, but they do provide a qualitative guide to the type of transitions in relatedness among taxa that occurs between depth strata.

Second, we examined the effect of excluding specific taxa from the calculation of AvTD and VarTD [53]. After visually examining the taxonomic trees, we identified several taxa of interest. We then re-calculated AvTD (or VarTD) excluding the individual taxon of interest and subtracted these values from the AvTD for the full data set (e.g., AvTD - AvTD_no taxon 1_ = AvTD_diff_). While one could examine only AvTD_no taxon 1_, AvTD_diff_ givers a better visual interpretation of the effect of that taxon on the overall AvTD. For each taxon of interest, we analysed the relationship between AvTD_diff_ vs. depth and latitude using GAMs as described above. In these analyses, negative values of AvTD_diff_ indicate that the presence of that taxon decreased AvTD in the original analysis of all data because AvTD increased when it was removed, while positive values indicate the opposite.

## Results

We examined 1948 trawls from surveys conducted between 1999 and 2003. These trawls contained 243 species from 75 families, 30 orders and four classes. The most speciose families were Scorpaenidae (rockfishes, 48 spp.), Pleuronectidae (righteye flounders, 16 spp.), Zoarcidae (eelpouts, 14 spp.), Cottidae (sculpins, 15 spp.), Liparidae (snailfishes, 11 spp.) and Rajidae (skates, 8 spp.) The most speciose orders were Scorpaeniformes (mail cheeked fishes including rockfishes and scorpion fishes, 90 spp.), Perciformes (perches, 34 spp.), Pleuronectiformes (flatfishes, 21 spp.), Gadiiformes (cods, 13 spp.), Argentiniformes (marine smelts, 11 spp.), and Stomiiformes (dragonfishes, 11 spp.). By class the majority of fishes were Actinopterygiians (ray-finned fishes, 218 spp.) or Chondrichthyians (cartilaginous fishes, 23 spp.) with one Myxini (hagfishes) and one Petromyzontida (lampreys).

### Taxonomic Distinctness, depth and latitude

**Figure 2:**
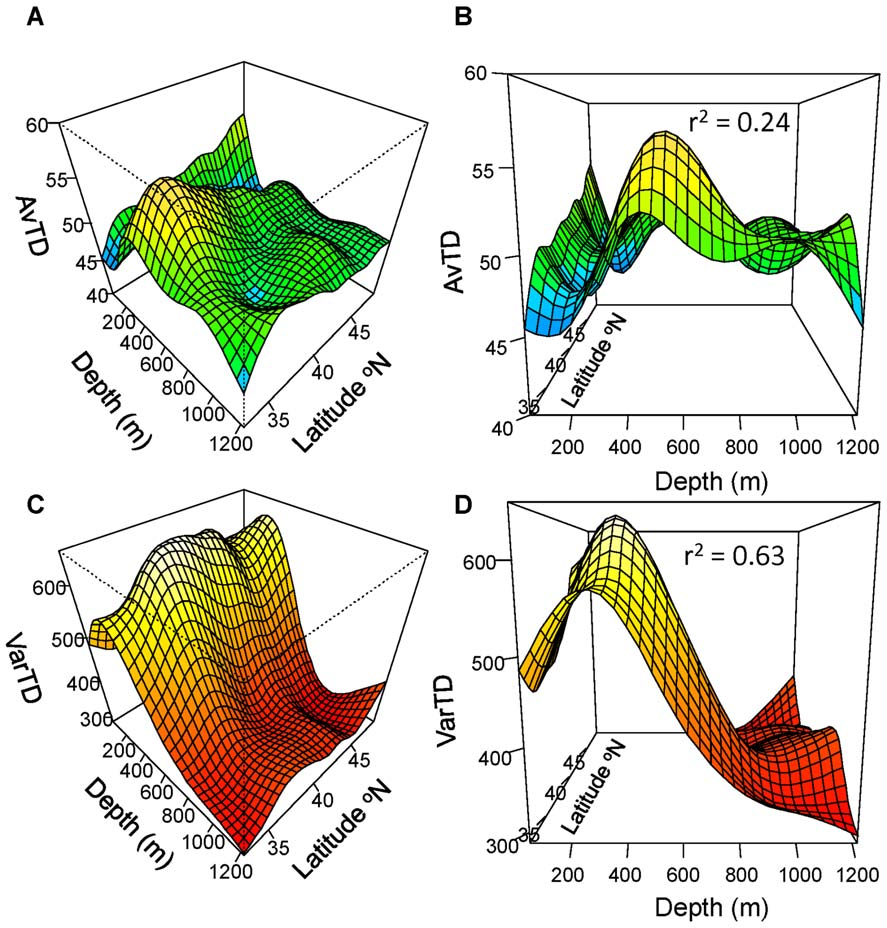
Taxonomic distinctness versus latitude and depth. (A,B) two views of average taxonomic distinctness (AvTD), and (C,D) two views of the variation in taxonomic distinctness (VarTD). Data were analyzed with Generalized Additive Models.

Average taxonomic distinctness (AvTD) varied significantly with depth (F_7.61_ = 20.73, p<0.001) and latitude (F_7.84_ = 8.05, p<0.001), and there was also a significant depth x latitude interaction (F_13.38_ = 11.26, p<0.001)(Fig. 2a,b). The GAM explained 24% of the variation in AvTD (r^2^ = 0.24, n = 1948). AvTD was highest around 500 m, especially in the region around 35°N. AvTD was lowest around 200 m and intermediate at depths over approximately 800 m. AvTD in the shallowest regions was similar to that found in the deepest depths. The estimated intercept (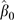) was 49.34 (±0.09 s.e.) indicating that, on average, species were related between the level of Superorder and Subdivision (Table 1).

Variation in taxonomic distinctness (VarTD) was also significantly affected by depth (F_5.25_ = 65.24, p<0.001), latitude (F_7.56_ = 3.14, p = 0.002), and their interaction (F_12.16_ = 10.78, p<0.001)(Fig. 2c,d). The GAM explained 63% of the variation in VarTD (r^2^ = 0.63, n = 1948). VarTD generally increased from the shallower areas, reaching a peak around 200–300 m and then decreasing sharply with increasing depth. Overall, there was a weak correlation between AvTD and VarTD (n = 1946, r = 0.27, p<0.001).

To further investigate depth x latitude interactions, we established five depth zones based primarily on the AvTD patterns in Figure 2: 50–150 m, 151–300 m, 301–600 m, 600–900 m, 900–1220 m. The shallowest depth bin was limited to the shelf and was set to encompass depths at which AvTD appeared to increase with increasing latitude, in contrast to the rest of the data. The 151–300 m zone brackets an area where AvTD is lowest (around 200 m). It includes the deepest portions of the shelf and the shallow areas of the slope. AvTD was highest at depths between 301–600 m and decreased between 601–900 m. It then appeared to remain fairly stable at depth. For consistency, we utilized these same depth bins for VarTD.

**Figure 3:**
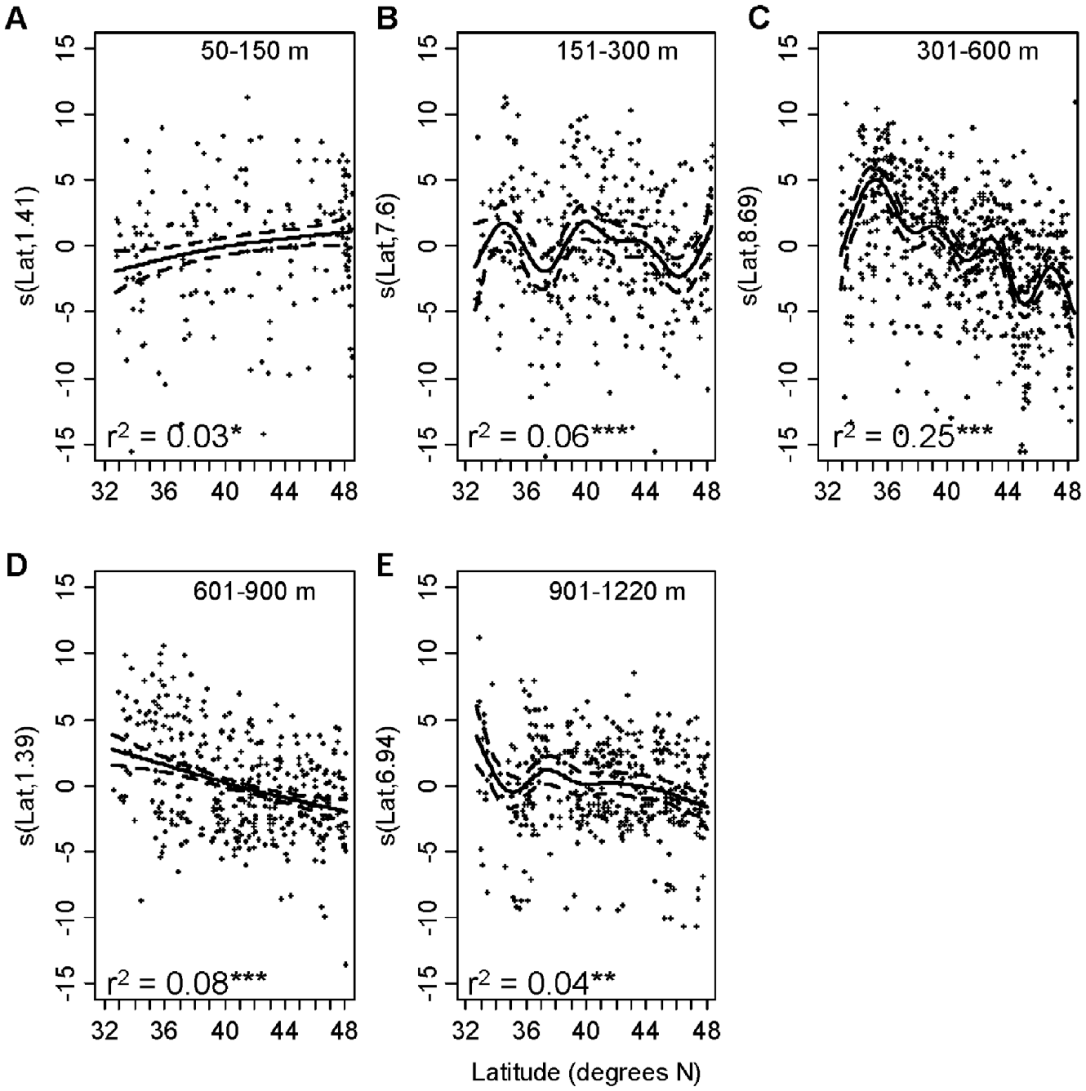
Average taxonomic distinctness versus latitude for five depth zones. Data were analyzed with Generalized Additive Models. Solid lines represent the smoothed trend. Broken lines are ±1 s.e. The data points shown are the residuals around the smoothed term. The y-axis label is the smoothed parameter and its estimated degrees of freedom from the GAM. Trends for the 50–150 m and 601–900 m depth bins were better represented by linear models. *  = p<0.05, **  = p<0.01, ***  = p<0.001.

When binned by depth, latitude explained significant but small amounts of variation in AvTD at all depths (Fig. 3). In the shallowest depth zone (50–150 m), a linear model was sufficient (analysis of deviance, p = 0.12) to describe the relation between AvTD and latitude. AvTD increased with latitude but the explained variance was just over three percent (linear regression, F_1, 169_ = 6.26, p = 0.017, r^2^ = 0.033). Between 151–300 m the relationship between AvTD and latitude was more complex with peaks in AvTD around 35°N and 40°N, and with lows around 32°N, 37°N and 45–46°N, but with no overall trend. The effect of latitude on AvTD was strongest in the 301–600 m depth zone with latitude explaining 25% of the variance in AvTD. AvTD peaked just north of 35°N and then declined overall as latitude increased, with a number of peaks and troughs. At 601–900 m a linear trend was sufficient (analysis of deviance, p = 0.13) to describe a decrease in AvTD with latitude but explained only 7% of the variation (linear regression, F_1, 381_ = 31.91, p<0.001, r^2^ = 0.07). In the deepest area (901–1220 m), AvTD decreased rapidly between 32°N and 35°N. It then increased until approximately 37–38°N after which it decreased with increasing latitude.

**Figure 4:**
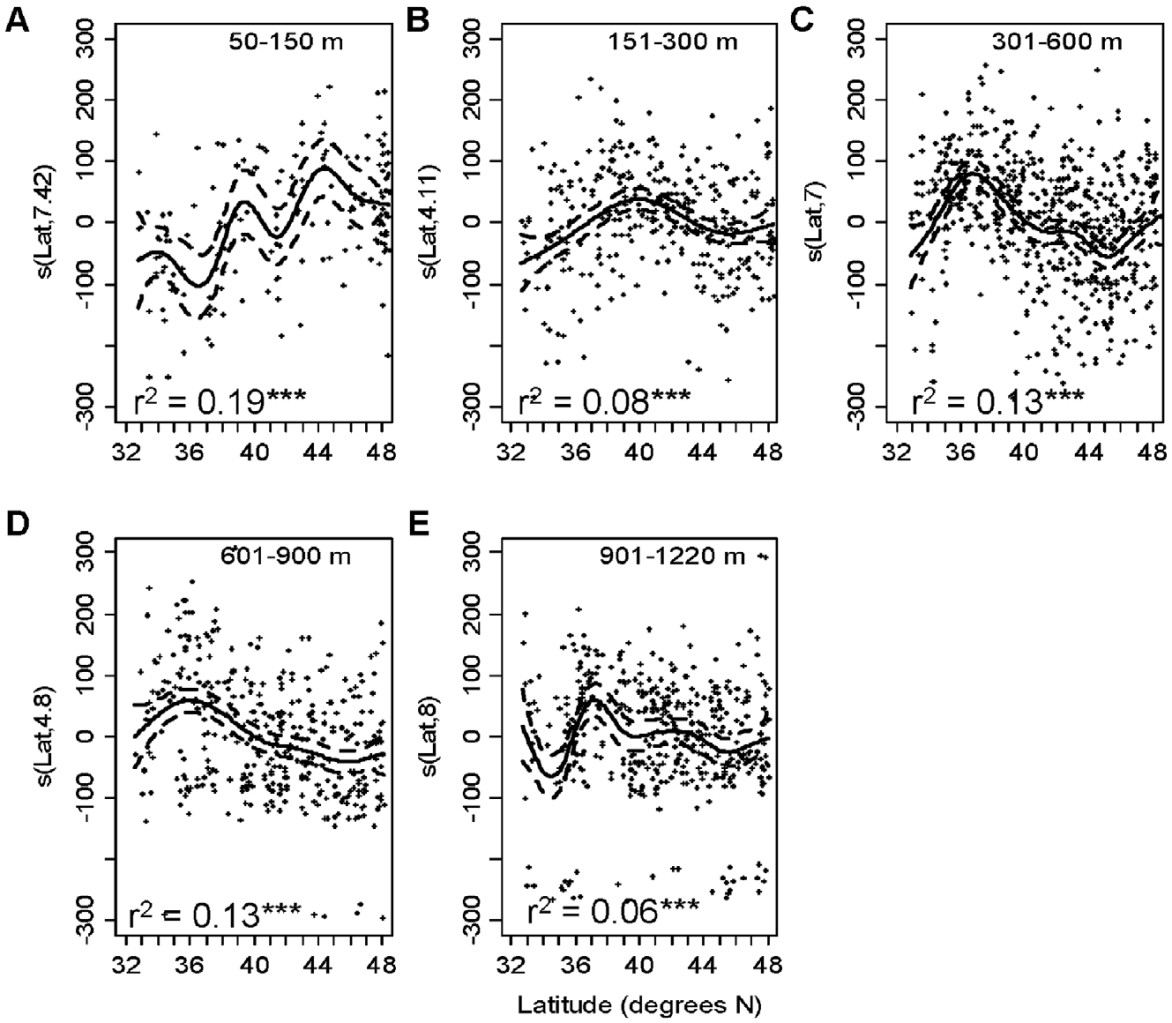
Variation in taxonomic distinctness versus latitude for five depth zones. Data were analyzed with Generalized Additive Models. Solid lines represent the smoothed trend. Broken lines are ±1 s.e. The data points shown are the residuals around the smoothed term. The y-axis label is the smoothed parameter and its estimated degrees of freedom from the GAM. *  = p<0.05, **  = p<0.01, ***  = p<0.001.

VarTD was also only weakly related to latitude within the five depth strata (Fig. 4). At 50–150 m, VarTD increased with latitude, explaining 19% of the variance, with peaks at approximately 34°N, 39°N and 45°N. In the 151–300 m depth zone, latitude explained only 8% of the variance in VarTD. VarTD increased from 32°N to 40°N and then decreased to the north. At 301–600 m VarTD increased between 32°N and approximately 37°N and then decreased to a low at 45°N. At 601–900 there was a similar peak in VarTD around 36–37°N. At 900–1220 m, VarTD was lowest between 34–35°N and peaked around 38°N, but there was no clear overall trend with latitude.

**Figure 5:**
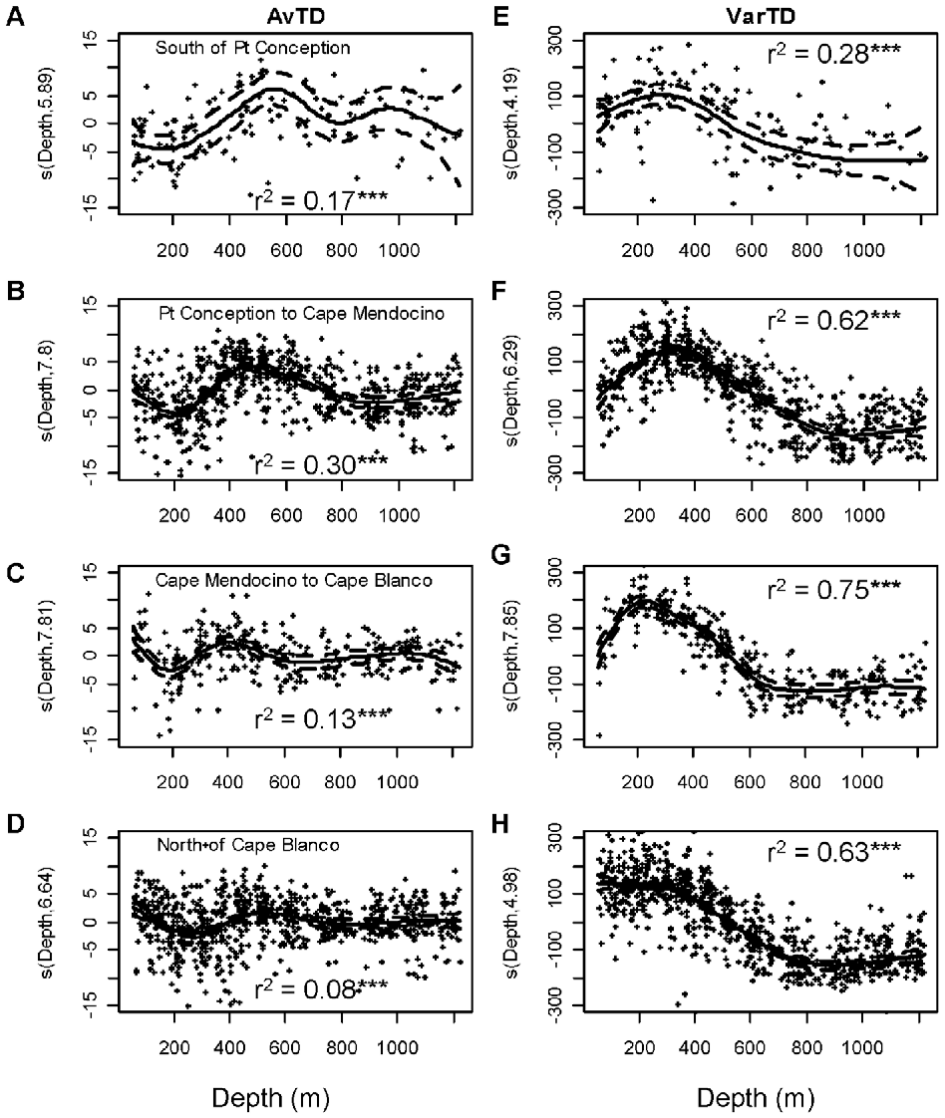
Taxonomic distinctness versus depth for different latitude zones. (A–D) AvTD and (E–H) VarTD. Latitude zone given in panes (A–D) corresponds with panes (E–H). Data were analyzed with Generalized Additive Models. Solid lines represent the smoothed trend. Broken lines are ±1 s.e. The data points shown are the residuals around the smoothed term. The y-axis is the smoothed parameter and its estimated degrees of freedom from the GAM. *  = p<0.05, **  = p<0.01, ***  = p<0.001.

The relationship between either AvTD or VarTD and depth was stronger (higher r^2^) than their respective relationships with latitude (Fig. 5). The explained variance for AvTD was highest in the Point Conception to Mendocino latitude bin and fairly low elsewhere. For VarTD, the variance explained by depth was much higher, being greater than 62% in three of the four latitude bins. There was some variation, as expected from the significant latitude x depth interaction terms in the main GAMs, with peak values for both AvTD and VarTD shifting among latitude bins.

### Comparison of taxonomic trees

**Figure 6:**
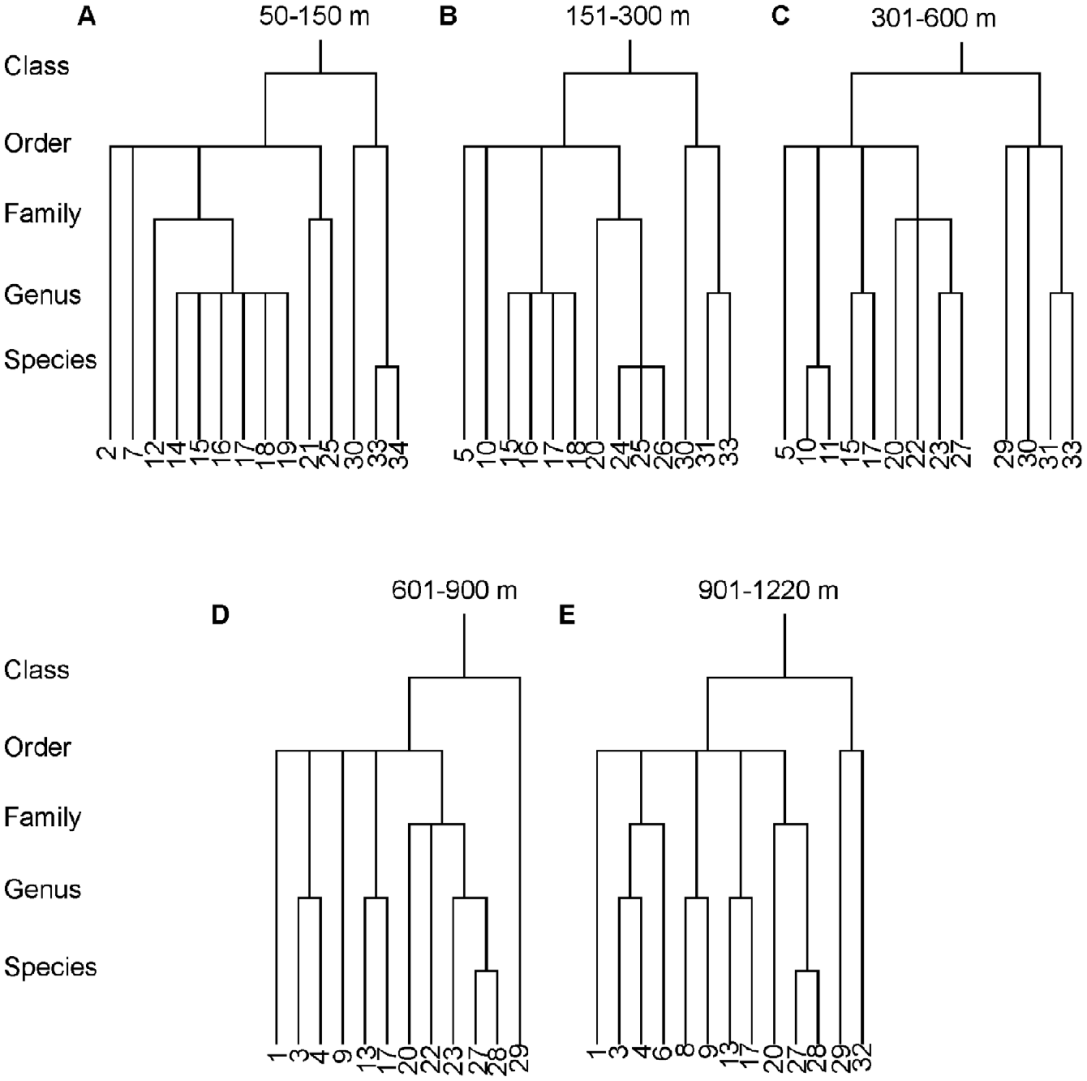
Taxonomic trees for the region between Point Conception and Cape Mendocino for five depth zones. Species are those found in at least 50% of the trawls in a given depth zone. Numbers indicate species identified in Table 2.

The taxonomic trees provided here, consisting of those species found in at least 50% of the trawls within a given depth bin, comprised 12–14 species per depth bin for the region between Point Conception and Cape Mendocino. Thirty-four species were found at least 50% of the time in at least one depth bin (Fig. 6, Table 2). Several taxa (Pleuronectiformes, Scorpaeniformes and Chondrichthyes, in particular) showed differences in their branching patterns, which may help explain the variation in AvTD among depths.

**Table 2:** Taxonomic information for species in Figure 6.

Class	Order	Family	Genus	Species	Common Name	ID
Actinopterygii						
Argentiniformes		Alepocephalidae	*Alepocephalus*	*tenebrosus*	California slickhead	1
Batrachoidiformes		Batrachoididae	*Porichthys*	*notatus*	plainfin midshipman	2
Gadiiformes		Macrouridae	*Albatrossia*	*pectoralis*	giant grenadier	3
			*Coryphaenoides*	*acrolepis*	Pacific grenadier	4
		Merlucciidae	*Merluccius*	*productus*	Pacific hake	5
		Moridae	*Antimora*	*microlepis*	Pacific flatnose	6
Perciformes		Embiotocidae	*Zalembius*	*rosaceus*	pink sea perch	7
		Zoarcidae	*Bothrocara*	*brunneum*	twoline eelpout	8
			*Lycenchelys*	*crotalinus*	snakehead eelpout	9
			*Lycodes*	*cortezianus*	bigfin eelpout	10
				*diapterus*	black eelpout	11
Pleuronectiformes		Paralichthyidae	*Citharichthys*	*sordidus*	Pacific sanddab	12
		Pleuronectidae	*Embassichthys*	*bathybius*	deepsea sole	13
			*Eopsetta*	*jordani*	petrale sole	14
			*Glyptocephalus*	*zachirus*	rex sole	15
			*Lyopsetta*	*exilis*	slender sole	16
			*Microstomus*	*pacificus*	Dover sole	17
			*Parophrys*	*vetulus*	English sole	18
			*Pleuronichthys*	*decurrens*	curlfin sole	19
Scorpaeniformes		Anoplopomatidae	*Anoplopoma*	*fimbria*	sablefish	20
		Hexagrammidae	*Ophiodon*	*elongatus*	lingcod	21
		Liparidae	*Careproctus*	*melanurus*	blacktail snailfish	22
		Scorpaenidae	*Sebastes*	*aurora*	aurora rockfish	23
				*diploproa*	splitnose rockfish	24
				*goodei*	chilipepper	25
				*saxicola*	stripetail rockfish	26
			*Sebastolobus*	*alascanus*	shortspine thornyhead	27
				*altivelis*	longspine thornyhead	28
Chondrichthyes						
Carcharhiniformes		Scyliorhinidae	*Apristurus*	*brunneus*	brown cat shark	29
Chimaeriformes		Chiamaeridae	*Hydrolagus*	*colliei*	spotted ratfish	30
Rajiformes		Rajidae	*Bathyraja*	*interrupta*	Bering skate	31
				*trachura*	roughtail skate	32
			*Raja*	*inornata*	California skate	33
				*rhina*	longnose skate	34

ID corresponds to the numbers on the taxonomic trees.

At 50–150 m, the high diversity of the Pleuronectidae (six species in six genera) appears to be counter-balanced by the relative paucity of species in other families or orders. The result is intermediate AvTD and high VarTD. The low AvTD around 200 m (here the 151–300 m depth stratum) appears to be due to a combination of the Pleuronectidae (four species in four genera) and the Scorpaeniformes (four species in two families), and, in particular, the genus *Sebastes*, which had three species. AvTD was highest between approximately 301–600 m. Some of the increase appears due to the addition of an entire order (Carcharhiniformes) to the Chondrichthyes at these depths. Additionally, there were only two Pleuronectids, and the Scorpaeniformes added families and genera while losing species within the *Sebastes*.

The primary change between the shallower areas and the deeper areas is the reduction of taxa within the Chondrichthyes in the zones deeper than 600 m. This likely explains much of the decrease in AvTD with depth as the number of species in different classes dropped. Additionally, the Scorpaeniformes gained a species (*Sebastolobus altivelis*). Among the deeper depth zones (those greater than 600 m), the Scorpaeniformes, Pluronectiformes, and Argentiniformes showed no change in branching structure. The continued decline in AvTD with depth appears to be due to the addition of a Morid, *Antimora microlepis* to the Gadiformes, and the addition of *Bothrocara brunneum* to the Zoarcidae.

The relationship between tree structure and VarTD is easy to discern in the current example. VarTD was highest in the shallower depth strata around 200 m in particular (Fig. 2c,d & 5e,f,g,h). At these depths, the trees contained a combination of short branches and long branches. There were high numbers of related species (four Pleuronectids and three *Sebastes*), but three of the four other orders contained only one species. Moreover four of the thirteen species were in the Chrondrichthyes and thus related to the remaining species at the level of class. At deeper depths, species were more evenly distributed among taxa with only one to two species per family (so fewer short path-lengths) and fewer Chondrichthyes (so relatively fewer long path-lengths).

### Influence of specific taxa on AvTD

**Figure 7:**
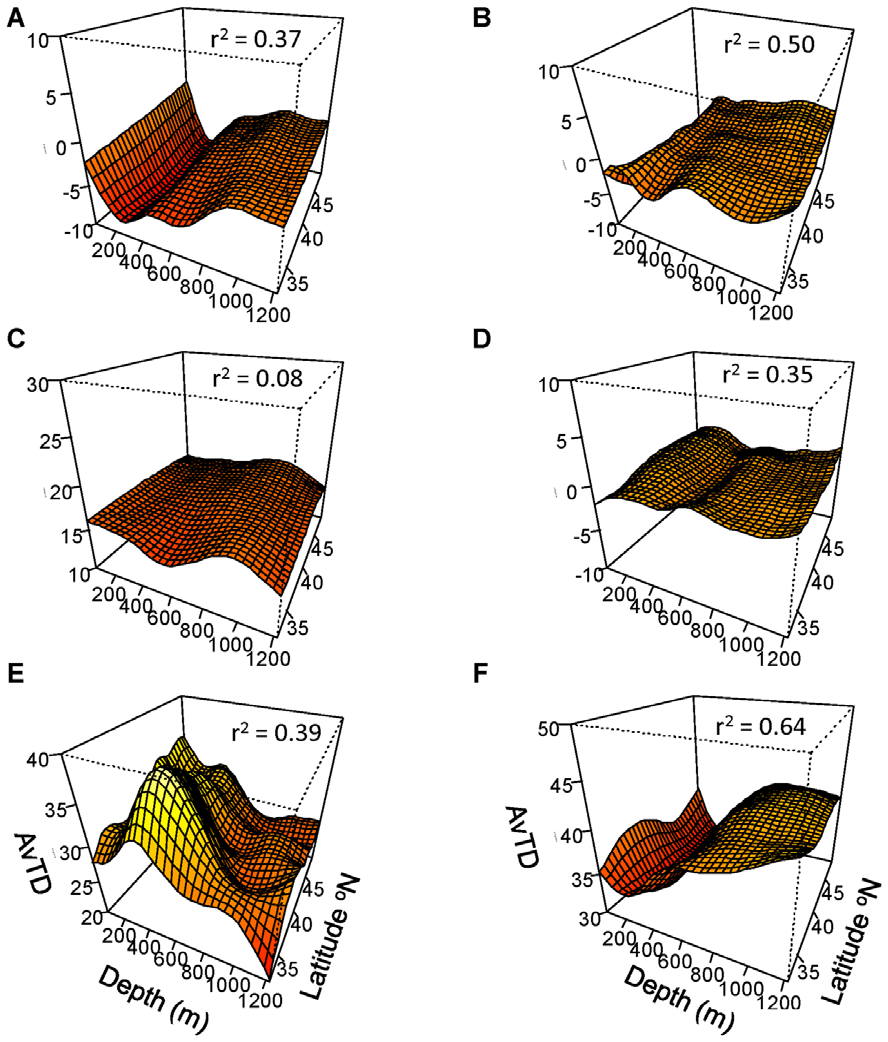
AvTD versus depth and latitude. AvTD_diff_ for (A) Scorpaeniformes, (B) Pleuronectiformes, (C) Perciformes, (D) Gadiformes, (E) Chondrichthyes, and (F) AvTD for Actinopterygii only. All GAMs are significant at the α = 0.05 level.

**Figure 8:**
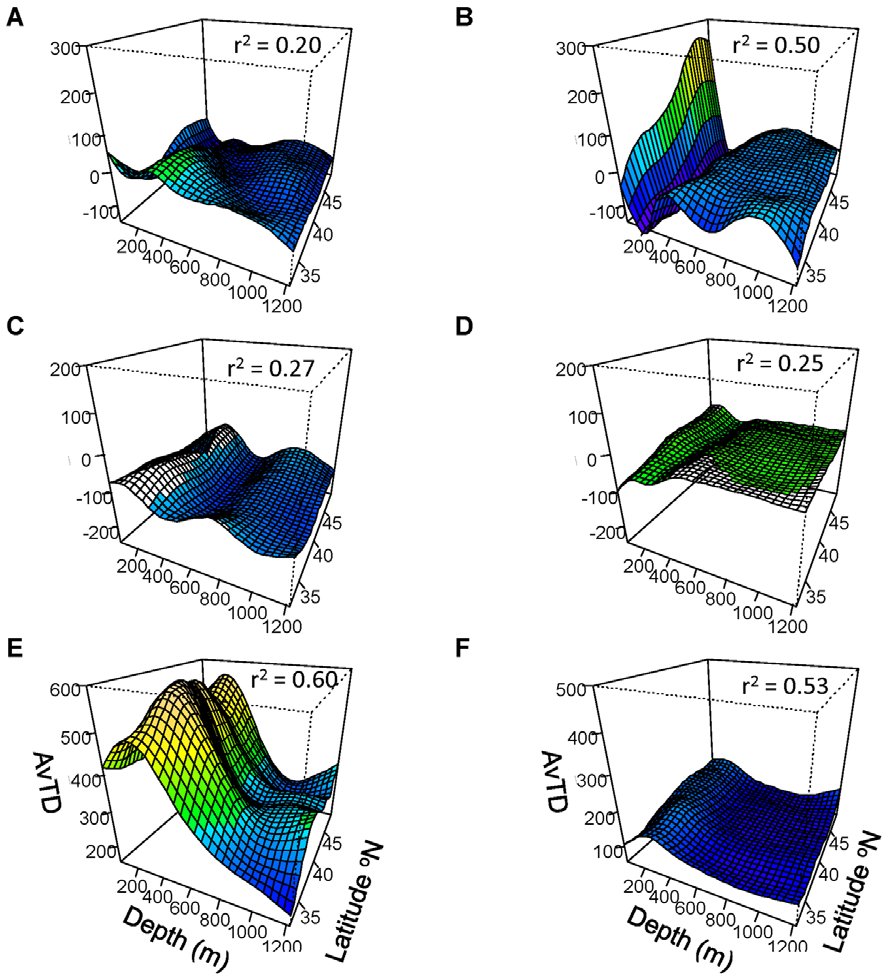
VarTD versus depth and latitude. VarTD_diff_ (A) Scorpaeniformes, (B) Pleuronectiformes, (C) Perciformes, (D) Gadiformes, (E) Chondrichthyes, and (F) VarTD for Actinopterygii only. All GAMs are significant at the α = 0.05 level.

Based on the analysis of the taxonomic trees, we chose to examine the effect on AvTD of removing five taxa: Scorpaeniformes, Pleuronectiformes, Gadiformes, Perciformes and the Chondrichthyes. AvTD_diff_ and VarTD_diff_ for all five taxa showed significant depth x latitude interactions (Fig. 7 & 8, GAM, p<0.05 for all). For brevity, we do not present full GAM results for each taxon but give only the r^2^ for each model.

AvTD_diff_ for Scorpaeniformes (Fig. 7a, r ^2^ = 0.37) was negative across all depths and latitudes indicating that this order generally lowered the AvTD for the full dataset. This was primarily due to there being many species in the genus *Sebastes*, as well as Scorpaeniformes being the most speciose order. AvTD_diff_ was lowest (ca. −10) at approximately 200 m across all latitudes, which coincides with the relatively high diversity of *Sebastes* at these depths seen in the tree analysis and the low overall AvTD at the same depth.

In regions deeper than approximately 200 m, removal of Pleuronectiformes (Fig. 7b, r ^2^ = 0.50) did not cause large changes in AvTD, which was negative in value but close to zero. However at depths shallower than 200 m, AvTD_diff_ was close to −10 indicating that this order lowered AvTD in the shallower areas. This pattern corresponds directly with the diversity of the flatfishes seen in the tree structure, with four to seven species found to occur in the shallower two zones but only two species in the deeper areas. AvTD_diff_ for the Pleuronectiformes also appeared to decrease with latitude in the shallows.

AvTD_diff_ for both Gadiformes (Fig. 7c, r ^2^ = 0.08) and Perciformes (Fig. 7d, r ^2^ = 0.35) also showed significant relationships with latitude and depth. The Gadiformes raised AvTD by 15 points or more overall, but showed only small differences in relation to depth and latitude. The Perciformes also showed only minor variation with depth and latitude, generally having only a small effect on AvTD (with AvTD_diff_ values near zero).

Removal of the class Chondrichthyes from the calculation of AvTD resulted in the most dramatic effects (Fig. 7e, r ^2^ = 0.39). AvTD_diff_ for Chondrichthyes was large and positive across all depths ranging from 20 to just under 40 and indicating that this taxon raised AvTD substantially in all areas. More importantly, AvTD_diff_ for the Chondrichthyes matched the overall pattern of AvTD from the full data set indicating that this taxon drove the general pattern of taxonomic distinctness. It was highest between about 300–600 m and declined with latitude in this depth region. It also increased with increasing latitude in the shallower areas and was lower and relatively flat at depth. These results match the tree branching patterns with the highest Chondrichthyan diversity seen in the 300–600 m depth areas and the lowest seen at depth.

AvTD calculated for only the Actinopterygii shows a substantially different pattern from the overall pattern obtained using the full data set (Fig. 7f). AvTD_Actin._ is still lowest around 200 m depth but is higher at depths greater than 600 m than in shallower regions. The depth x latitude interaction was significant (GAM, F_13.1_ = 4.40, p<0.001), as were both main effects of depth (F_6.9_ = 57.50, p<0.001) and latitude (F_4.5_ = 2.82, p = 0.02). The GAM showed that depth and latitude explained 64% of the variation in AvTD_Actin_. (n = 1948, r^2^ = 0.64).

For VarTD, removal of Scorpaeniformes (Fig. 8a, r ^2^ = 0.20), Pleuronectiformes (Fig. 8b, r ^2^ = 0.50), Gadiformes (Fig. 8c, r ^2^ = 0.27), and Perciformes (Fig. 8d, r ^2^ = 0.25) resulted in negative VarTD_diff_ values at most depths and latitudes, indicating that these taxa generally lowered VarTD when included in the analysis. The Scorpaeniformes did increase VarTD (positive VarTD_diff_ values) at approximately 200 m in the more southern areas, as did the Pleuronectiformes in the shallowest areas at higher latitudes. VarTD_diff_ for the exclusion of Chondrichthyes (r^2^ = 0.60), however, was strongly positive and generally matched the overall pattern of VarTD.

VarTD for the Actinopterygii was related to depth (F_7.12_ = 39.0, p<0.01), latitude (F_7.74_ = 4.0, p<0.01) and there was a significant depth x latitude interaction (F_11.3_ = 9.2, p<0.01) (Fig. 8f). The model explained 53% of the variation in VarTD for the Actinopterygii (r^2^ = 0.53, n = 1948). As for VarTD calculated using all taxa, VarTD for the Actinopterygii was highest around 200 m and decreased with depth.

## Discussion

Large-scale gradients in taxonomic distinctness have not been extensively studied in the marine environment. Here, taxonomic distinctness (AvTD and VarTD) varied with both latitude and depth, but the latitude patterns were weak overall. This result is similar to other work in marine systems where trends of AvTD within phyla tend to be weak in relation to latitude (when controlling for depth) but variation with depth is stronger. For both latitude and depth, the two taxonomic distinctness measures showed patterns that differed from more traditional diversity measures, specifically species richness and evenness, for the same fish assemblage. Thus, AvTD and VarTD provide a perspective on diversity that differs somewhat from that obtained when analyzing richness or evenness alone. These distinctness measures provide important, complimentary information that should be of interest to biologists and managers. An important caveat to our analyses is that they are limited to trawl-sampled demersal fishes on primarily soft bottoms where trawl surveys can be conducted. Patterns on hard, complex substrata (e.g., rocky reefs) or with fishes not susceptible to capture by trawls may differ.

### Latitude and Depth Trends

The relationship between taxonomic distinctness and either latitude or depth for various marine taxa has not been extensively studied. Where it has been investigated, the relationship with latitude is generally weak and relationships with depth are stronger [27], [39], [41], [54], although this weak latitude relationship is not always obvious unless one controls for depth [54]. For annelids and crustaceans on the Norwegian continental shelf, and for macrobenthos on the European continental shelf, AvTD increases with increasing latitude but the explained variation is small [39], [54]. However north-east Atlantic demersal fishes do show geographic variation in taxonomic distinctness between the western waters of the United Kingdom and the southern North Sea, with Elasmobranchs contributing substantially to the patterns [53]. At very large spatial scales, AvTD for pelagic copepods shows low variability between 40°N and 40°S but then becomes highly variable as it declines towards the poles [55].

Latitudinal trends in both AvTD and VarTD were present in west coast demersal fishes but were generally very weak. On the shelf (here the 50–150 m depth stratum) both AvTD and VarTD increased with latitude, as seen with macrobenthos on the Norwegian and European shelves. While the relationship was very weak for AvTD and only moderately strong for VarTD, in both cases these trends are the reverse of the typical trend of diversity (in the sense of species richness), which decreases with increasing latitude. The only moderately strong latitudinal trend was for AvTD between 301–600 m, where latitude explained 25% of the variation. In the deeper areas, AvTD and Var TD tended to decrease with increasing latitude following the more common diversity-latitude pattern.

Latitudinal trends in taxonomic distinctness for demersal fishes on the continental slope (here deeper than 151 m, although this does include a small portion of the shelf) differed from those reported for species richness and evenness [23], especially in the more shallow areas of the slope (Tolimieri [23] did not calculate richness or evenness values for the shelf, so we cannot make comparisons with results obtained from the 50–150 m depth bin). Richness and evenness were both positively correlated with latitude between 150–349 m and richness between 350–549 m [23]. However, AvTD varied but showed no overall trend with latitude between 151–300 m, and it was negatively correlated with latitude between 301–600 m. In deeper areas, all metrics tended to show a decline with latitude.

Relationships between each of the two distinctness measures and depth were stronger (higher r^2^) than they were with latitude — especially for VarTD. Perhaps the most interesting pattern relates to the 151–300 m depth stratum. Richness and evenness for slope demersal fishes [23] at similar depths were both relatively high, as was VarTD in the present study. However AvTD was relatively low in this depth stratum, giving a different perspective on the nature of diversity. Combining the different diversity measures, we can conclude that there were many, closely related species at these depths, in particular within the family Scorpaenidae.

In deeper areas, richness, evenness and AvTD showed a general decrease with depth from high values around 301–600 m (400–500 m stratum in Tolimieri [23]), while species density and VarTD peaked and declined at shallower depths, generally around 200 m. Intermediate to low diversity at depth was due to several factors: fewer species, a less even representation of those species in terms of their relative abundances, higher relatedness among those species and a more consistent branching pattern in the classification tree — the latter two features being due to there being fewer Chondrichthyes. However, within the Actinopterygii, AvTD was actually higher at depth than in more shallow areas. High Actinopterygiian AvTD at depth was due to these species being more evenly distributed among orders. This strong effect of cartilaginous fishes on taxonomic distinctness measures was also seen by Rogers et al. [53] for demersal fishes in the waters from the western United Kingdom to the southern North Sea. Mechanistically or perhaps mathematically, the long branch lengths that the Chondrichthyes add (due to being related to most other taxa only at the level of class) and the variability in their frequency of occurrence, especially with depth, is the root of their strong effect on taxonomic distinctness patterns.

While patterns with depth were stronger than with latitude, in all cases the depth x latitude interaction was significant for AvTD and VarTD. This result is similar to that seen for both assemblage structure [56] and species richness and evenness trends [23] in west coast demersal fishes. For example, northerly trawls had deeper/colder-water assemblages at a given depth than did sites farther to the south [56], although this pattern disappeared at deeper areas. Bottom temperatures decrease with increasing latitude, especially in the shallower areas of the slope, and are a potential cause of the depth x latitude interaction.

The low explained variation for AvTD (∼25%) may result from a general lack of any strong pattern at this spatial scale or from missing explanatory variables, with substratum type being an obvious potential factor. Diversity can differ among habitat types and more complex combinations of multiple habitat types often leads to higher diversity in terms of species richness [57]. While taxonomic distinctness may differ naturally among habitats, the effect does not seem to be strong, at least among soft bottom communities (e.g., mud versus sand). For example, sediment grain size explained little variation in AvTD for annelids, crustaceans and molluscs on the Norwegian Shelf. The greatest variance explained by sediment grain size was around 2.1% for crustaceans. Similarly, unless sites are degraded somewhat, AvTD for free-living nematodes [58] and for megafauna, macroinfauna and nematodes [59] tends to be similar to expectations based on the regional species pool.

The data analyzed here did not include samples from complex, rocky habitat where towing nets is difficult or impossible. As such, the largest potential difference in habitat type (rocky versus soft sediment) did not influence the analyses. The importance of rockfish to AvTD, especially around 200 m, suggests that habitat might have a strong effect on AvTD if complex, rocky habitats were included in the analyses. Rockfish are very diverse, with 72 species in the northeast pacific, and they are most common in rocky habitats [60]. One might expect, therefore, that rocky habitats dominated by many closely related species (with lots of rockfish) would have low AvTD compared to other areas with an assemblage more diverse at higher taxonomic levels.

There is variation in oceanic habitat (upwelling intensity, chlorophyll-a, temperature, salinity, etc.) along the west coast that correlates with the assemblage structure of demersal fishes [61]. Similarly, assemblage structure and diversity patterns of oceanic copepods appear to be related to productivity regimes [55]. These oceanic habitats might help to explain some of the large-scale patterns seen in our data. For example the peak in AvTD in the 50–150 m depth bin between approximately 43–46 °N corresponds with oceanic habitat characterized by consistent upwelling [61]. However large-scale changes in oceanic habitats alone would not be enough to explain the rather large spread of residuals (unexplained variation) around the trend lines in our analyses.

### Biogeography

On the west coast of the US there are a number of geological features that may be important as biogeographic boundaries or transition zones. Point Conception (∼34.5°N) has long been thought to be a boundary for fishes [62]. However, more recent phylogeographic [63] and range end-point analyses [64] have suggested that the Los Angeles Region and Monterey Bay Region, both of which have submerged canyons, are the more likely boundaries for fishes [63], molluscs [65], [66] and marine algae [67]. Point Conception is better interpreted as a transition zone as opposed to a barrier [63], and there is some evidence that this transition zone may have shifted over time [64]. For slope demersal fishes, the assemblage structure [56], species richness and evenness [23] all showed some relation to geographic features, although there is variability among depth zones. In terms of assemblage structure, Cape Mendocino was the most consistent region of change in the assemblage structure of the 26 most abundant slope demersal fishes. Richness tended to show highs around Point Conception and lows near Monterey Bay, while evenness tended to be low around Cape Mendocino.

While the patterns vary somewhat among the depth zones, AvTD and VarTD tended to show peaks around Point Conception and Cape Mendocino and showed both lows and highs around the Monterey Bay area (ca. 37–38°N). There was some variation with depth, as Point Conception tended only to be important in the more shallow depth strata. The high diversity in most metrics around Point Conception makes sense if it is a transition zone between northern and southern fauna [62]–[64]. Given the general weakness of latitudinal trends in taxonomic distinctness, these observations should not be overly stressed.

### Biodiversity, conservation and management

Conservation and management efforts often focus on maintaining biodiversity, specifically species richness [12], [14], [68]. At one level this focus is an attempt to protect as many species as possible with limited resources [69]. At another level, the potential relationship between ecosystem function and biodiversity also makes the maintenance of richness a priority [6], [8]–[10].

Diversity is, however, a complex idea, and it can be measured in a number of complimentary ways [28]. While important and essential to our understanding of communities, measures like species richness and evenness do not recognize the taxonomic relationships among species, and treat all species as equivalent. More recently a number of metrics have been developed that do take into consideration the evolutionary relationships, including taxonomic diversity [70], phylogenetic diversity [71], [72] and the taxonomic distinctness measures (AvTD and VarTD) used here. The fact that these taxonomic distinctness measures are statistically independent of either the sampling intensity or richness of samples [30], [32] makes them especially attractive tools for investigating structural biodiversity.

Taxonomic distinctness often provides complimentary or contradictory information to the more typical richness analyses. For example, species richness was lower in the Columbretes Island Marine Reserve than in reference sites. However, taxonomic distinctness was higher in the reserve [34]. For demersal fishes on the slope in the present study, richness and evenness were highest in the shallow portion of the continental slope, but this area had low AvTD due to the high relatedness of species (mostly rockfish or flatfish) in this depth stratum. In fact, for the Actinopterygii, diversity measured as AvTD was the opposite of overall richness patterns, being highest at depth.

Focusing on species richness alone does not recognize diversity among deeper evolutionary lineages. The presence of several distinct lineages at higher taxonomic levels may be important to provide resources for future evolutionary innovation [69], [73], especially since extinction and speciation rates differ among taxa. Measures of taxonomic distinctness may also represent functional diversity to some extent [74]. Not evaluating diversity at higher taxonomic levels fails to recognize potentially important patterns of diversity. For example, there are fewer plant species in Malesia—a region including Malaysia to Papua New Guinea and the Solomon Islands—but there are more plant families in this region than in the rest of the neotropics. This is important since the number of families may provide a better measure of both functional diversity and the evolutionary potential of the assemblage [73]. This pattern would likely be reflected by higher AvTD values for Malesia than for other areas, as more species are related at higher taxonomic levels. We do not mean to suggest that metrics like species richness should be ignored, but a more holistic analysis of assemblage structure, including measures which incorporate the degree of relatedness among species, is necessary to fully understand patterns of diversity.

## Supporting Information

Figure S1 Residual plots for Generalized Additive Model (GAM) examining variation in AvTD versus depth and latitude. In the GAM, *y_i_* = β_0_ + *f*_1_(*x*_1*i*_) + *f*_2_(*x*_2*i*_) + *f*_12_(*x*_1*i*_×*x*_2*i*_) + ε*_i_* where *y*_i_ was AvTD, *x*_1_ was depth, *x*_2_ was latitude, β_0_ was the intercept parameter and ε_i_ were random normal errors with zero mean and a common variance. The smoothing functions *f*_1_ and *f*_2_ were thin plate regression splines [49]. Because depth and latitude were measured on different scales, we used a tensor product smooth (*f*_12_) of thin plate regression splines for the interaction term [51]. The optimal level of smoothing was chosen with general cross validation. Data were fit in R v2.10.0 using the package ‘mgcv’ [49], [52].
(0.20 MB PDF)

Figure S2 Residual plots for Generalized Additive Model (GAM) examining variation in VarTD versus depth and latitude. In the GAM, *y_i_* = β_0_ + *f*_1_(*x*_1*i*_) + *f*_2_(*x*_2*i*_) + *f*_12_(*x*_1*i*_ × *x*_2*i*_) + ε*_i_* where *y*_i_ was VarTD, *x*_1_ was depth, *x*_2_ was latitude, β_0_ was the intercept parameter and ε_1_ were random normal errors with zero mean and a common variance. The smoothing functions *f*_1_ and *f*_2_ were thin plate regression splines [49]. Because depth and latitude were measured on different scales, we used a tensor product smooth (*f*_12_) of thin plate regression splines for the interaction term [51]. The optimal level of smoothing was chosen with general cross validation. Data were fit in R v2.10.0 using the package ‘mgcv’ [49], [52].
(0.27 MB PDF)
