# Supplementary figures and images for: Taxonomic Distinctness of Demersal Fishes of the California Current: Moving Beyond Simple Measures of Diversity for Marine Ecosystem-Based Management
Source: PLoS One. 2010 May 17;5(5):e10653. doi: 10.1371/journal.pone.0010653 (PMC2871800; doi:10.1371/journal.pone.0010653)

**Normal Q-Q Plot**

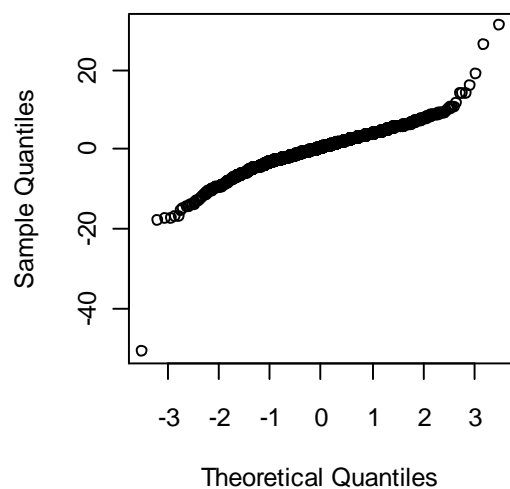

**Resids vs. linear pred.**

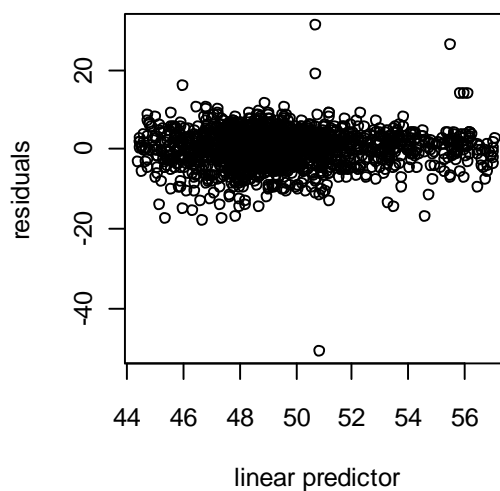

**Histogram of residuals**

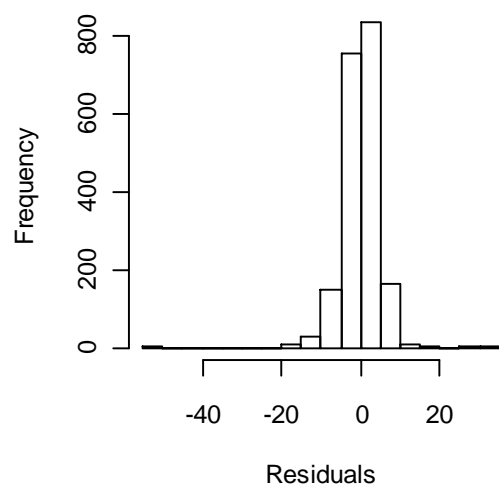

**Response vs. Fitted Values**

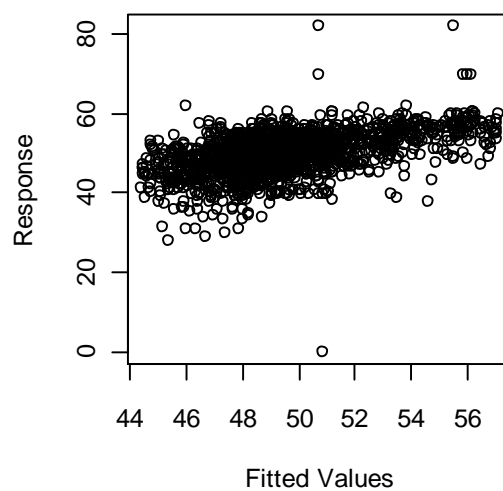

Supplement: Figure S1 — Residual plots for Generalized Additive Model (GAM) examining variation in AvTD versus depth and latitude. In the GAM, yi = β0 + f 1(x 1i) + f 2(x 2i) + f 12(x 1i×x 2i) + εi where y i was AvTD, x 1 was depth, x 2 was latitude, β0 was the intercept parameter and εi were random normal errors with zero mean and a common variance. The smoothing functions f 1 and f 2 were thin plate regression splines [49]. Because depth and latitude were measured on different scales, we used a tensor product smooth (f 12) of thin plate regression splines for the interaction term [51]. The optimal level of smoothing was chosen with general cross validation. Data were fit in R v2.10.0 using the package ‘mgcv’ [49], [52]. (0.20 MB PDF) [file pone.0010653.s001.pdf]

**Normal Q-Q Plot**

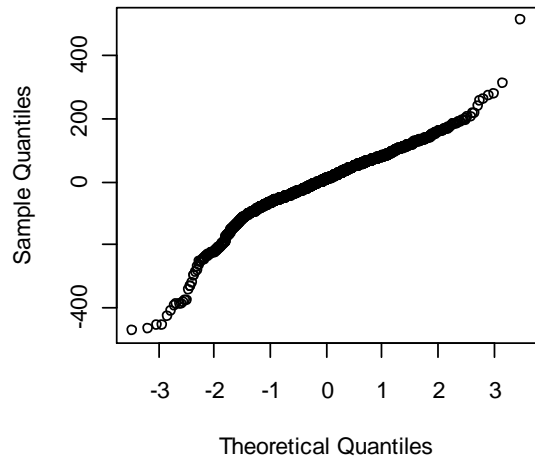

**Resids vs. linear pred.**

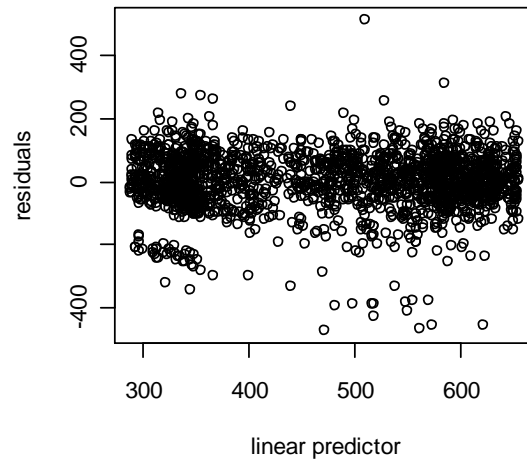

**Histogram of residuals**

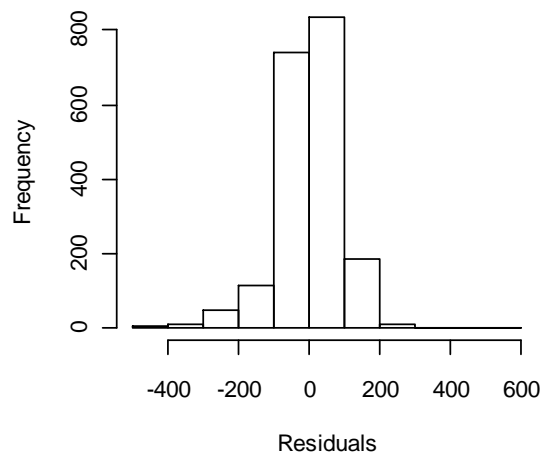

**Response vs. Fitted Values**

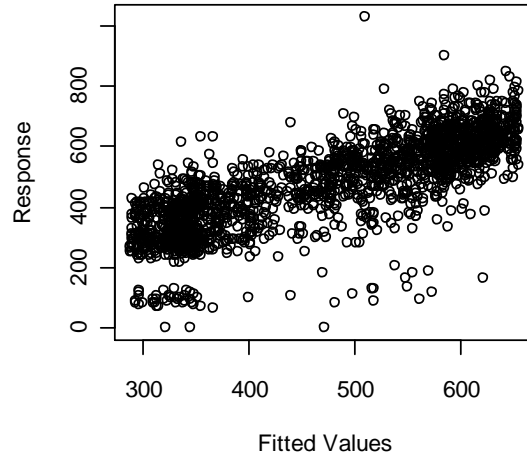

Supplement: Figure S2 — Residual plots for Generalized Additive Model (GAM) examining variation in VarTD versus depth and latitude. In the GAM, yi = β0 + f 1(x 1i) + f 2(x 2i) + f 12(x 1i × x 2i) + εi where y i was VarTD, x 1 was depth, x 2 was latitude, β0 was the intercept parameter and ε1 were random normal errors with zero mean and a common variance. The smoothing functions f 1 and f 2 were thin plate regression splines [49]. Because depth and latitude were measured on different scales, we used a tensor product smooth (f 12) of thin plate regression splines for the interaction term [51]. The optimal level of smoothing was chosen with general cross validation. Data were fit in R v2.10.0 using the package ‘mgcv’ [49], [52]. (0.27 MB PDF) [file pone.0010653.s002.pdf]
